# A ten N6‐methyladenosine‐related long non‐coding RNAs signature predicts prognosis of triple‐negative breast cancer

**DOI:** 10.1002/jcla.23779

**Published:** 2021-05-02

**Authors:** Jie Wu, Yan Cai, Gaiping Zhao, Maolan Li

**Affiliations:** ^1^ Key Laboratory of Hydrodynamics (Ministry of Education) School of Naval Architecture, Ocean and Civil Engineering Shanghai Jiao Tong University Shanghai China; ^2^ School of Biological Science and Medical Engineering Southeast University Nanjing China; ^3^ School of Medical Instrument and Food Engineering University of Shanghai for Science and Technology Shanghai China; ^4^ Shanghai Research Center of Biliary Tract Disease Shanghai China

**Keywords:** ceRNA network, long non‐coding RNA, N6‐methyladenosine, prognostic signature, triple‐negative breast cancer

## Abstract

**Background:**

Patients with triple‐negative breast cancer (TNBC) face a major challenge of the poor prognosis, and N6‐methyladenosine‐(m6A) mediated regulation in cancer has been proposed. Therefore, this study aimed to explore the prognostic roles of m6A‐related long non‐coding RNAs (LncRNAs) in TNBC.

**Methods:**

Clinical information and expression data of TNBC samples were collected from TCGA and GEO databases. Pearson correlation, univariate, and multivariate Cox regression analysis were employed to identify independent prognostic m6A‐related LncRNAs to construct the prognostic score (PS) risk model. Receiver operating characteristic (ROC) curve was used to evaluate the performance of PS risk model. A competing endogenous RNA (ceRNA) network was established for the functional analysis on targeted mRNAs.

**Results:**

We identified 10 independent prognostic m6A‐related LncRNAs (*SAMD12*‐*AS1*, *BVES*‐*AS1*, *LINC00593*, *MIR205HG*, *LINC00571*, *ANKRD10*‐*IT1*, *CIRBP*‐*AS1*, *SUCLG2*‐*AS1*, *BLACAT1*, and *HOXB*‐*AS1*) and established a PS risk model accordingly. Relevant results suggested that TNBC patients with lower PS had better overall survival status, and ROC curves proved that the PS model had better prognostic abilities with the AUC of 0.997 and 0.864 in TCGA and GSE76250 datasets, respectively. Recurrence and PS model status were defined as independent prognostic factors of TNBC. These ten LncRNAs were all differentially expressed in high‐risk TNBC compared with controls. The ceRNA network revealed the regulatory axes for nine key LncRNAs, and mRNAs in the network were identified to function in pathways of cell communication, signaling transduction and cancer.

**Conclusion:**

Our findings proposed a ten‐m6A‐related LncRNAs as potential biomarkers to predict the prognostic risk of TNBC.

## Graphical abstract

Our study firstly investigated the prognostic roles of m6A‐related LncRNAs in TNBC patients. In this study, ten m6A‐related LncRNAs were newly identified to be related with the prognosis of TNBC patients, and these candidate LncRNAs showed abilities to predict the prognostic risk of TNBC.

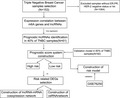

## 1 INTRODUCTION

Triple‐negative breast cancer (TNBC) is an aggressive subtype of breast cancer and is histochemically recognized by the negative expressions of estrogen receptor (ER), progesterone receptor (PR), and human epidermal growth factor receptor‐2 (HER‐2).^1^ TNBC contributes to about 10%–20% of breast cancer cases globally, with a disappointing survival prognosis.^2^, ^3^, ^4^ Due to the limitation of advanced progression on effective targeted drugs, chemotherapy is one of the remaining options for a systematic anticancer treatment,^5^ but the sensitivity to chemotherapy is an unavoidable difficulty. Furthermore, compared with other subtypes of breast cancer, patients with TNBC have higher risks of death after distant metastasis and local recurrence.^6^, ^7^ Considering the poor prognosis of patients with TNBC and the biocomplexity of TNBC, the identification of multi‐target predictors of prognostic response is in urgent.

N6‐methyladenosine (m6A) is the methylation that occurs at adenosine N6, which is the most abundant mRNA internal modification in eukaryotic cells.^8^ M6A methylation is dynamically reversible in mammalian cells, and its epigenetic modification is considered to regulate the self‐renewal, differentiation, invasion, and apoptosis of tumor cells by mediating the expression of cancer‐related genes.^9^ M6A can be installed by the methyltransferase complex known as writers, be removed by demethylase known as erasers, and be recognized by binding functional proteins know as readers, and the crosstalk among these three regulators is believed to involve in cancer growth and progression.^10^ Studies have proved that the overexpression of *ALKBH5*, an m6A eraser, could decrease the methylation of *NANOG* mRNA and increase the *NANOG* protein expression level, thus elevating the proportion of breast cancer stem cells.^11^ Niu et al. also identified *BNIP3* as an m6A‐related anti‐oncogene with negative correlation with *FTO* in expression level in breast cancer patients.^12^ In addition to effects of m6A methylation on mRNAs, studies also found its functional regulation in non‐coding RNAs.^13^, ^14^

Long non‐coding RNAs (LncRNAs) and microRNAs (miRNAs) are the main components of non‐coding RNAs involved in the regulation of genes at epigenetic, transcriptional, and post‐transcriptional levels, and also play crucial roles in cancer development and progression.^15^ Among them, LncRNAs act as competitive endogenous RNAs, can inhibit the function of miRNAs in tumor post‐transcriptional regulatory network of TNBC.^16^ A review also concluded that LncRNAs and m6A may play synergistic roles in cancer therapy.^17^ For instance, knockout of *METTL3* may reduce the m6A modification level of specific transcripts which results in the inactivation of LncRNA X chromosome, whereas *METTL3* was up‐regulated by LncRNA‐HBXIP which was highly expressed in breast cancer.^18^, ^19^ Although the effect of m6A on cancer and the mutual regulation mechanism between m6A and LncRNAs have been extensively studied, no m6A‐related LncRNAs have been identified to join in the prognosis of TNBC.

**FIGURE 1:**
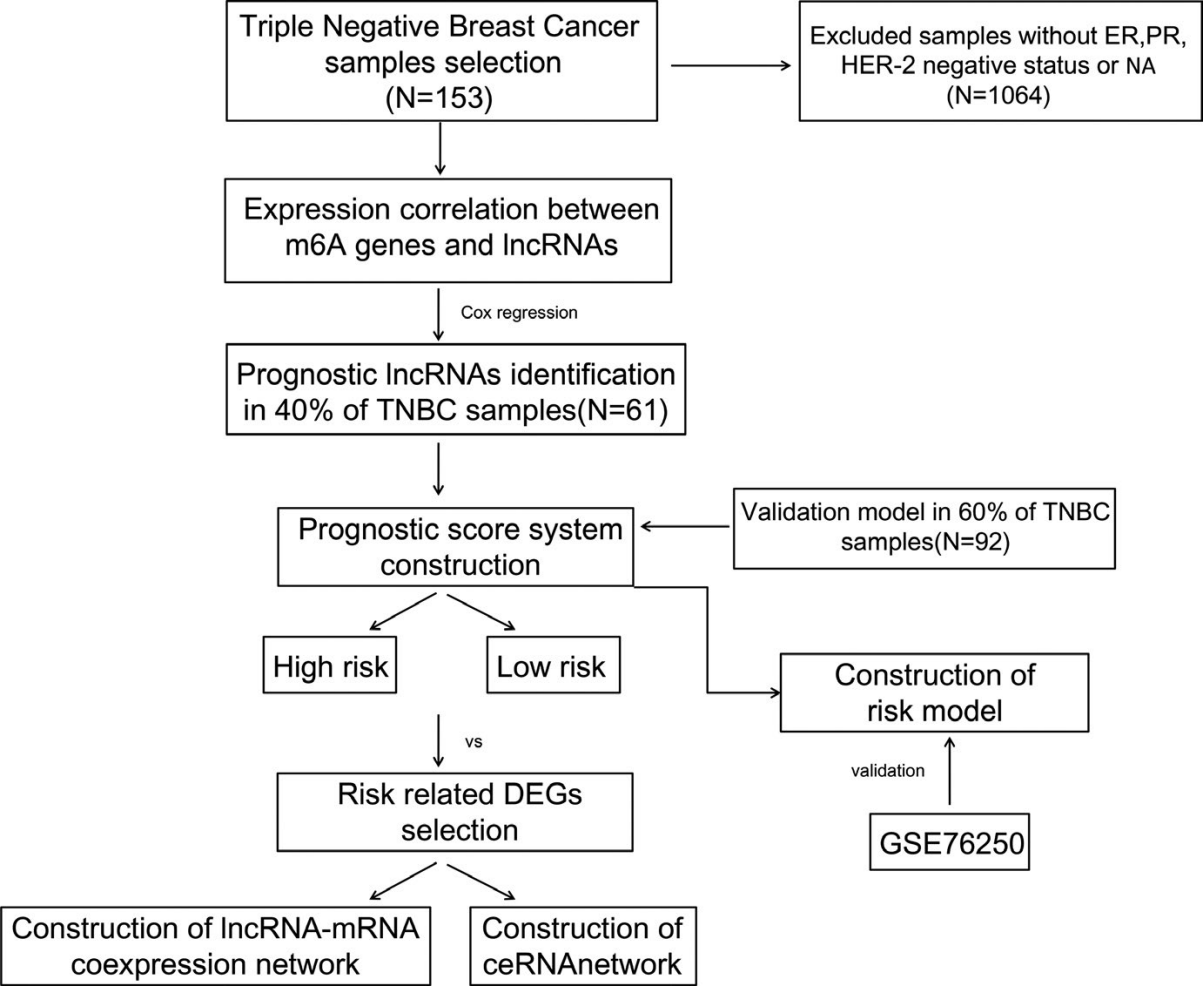
The flowchart of this study

Therefore, this study ascertained to screen m6A‐related LncRNAs in the expression level from TNBC samples and then to establish a prognostic risk model to evaluate the predictive abilities of candidate LncRNAs on TNBC prognosis. The procedures of this study were summarized and visualized in Figure 1. Our study will provide potential biomarkers for TNBC prognosis and help to improve treatment strategies for TNBC patients.

## 2 METHODS

### 2.1 Data acquisition and identification of m6A‐related LncRNAs

Expression data of RNA‐seq and clinical information of 153 TNBC samples (ER‐, PR‐ and HER‐2) was gathered from TCGA database, and GSE76250 dataset was screened out from NCBI‐GEO according to the following criteria: (1) has information to classify tumor subtypes; (2) has entity tumor sample tissues; (3) the sample size is over 100; and (4) more LncRNAs can be annotated from the testing platform.^20^, ^21^ We then annotated LncRNAs and mRNAs according to the corresponding annotation files of TCGA and GSE76250 and extracted expression data of m6A‐related genes (writers: *METTL3*, *METTL14*, *METTL15*, *WTAP*, *VIRMA*, *RBM15*, *RBM15B*, *KIAA1429*, *ZC3H13*; erasers: *FTO*, *ALKBH5*; readers: *RBMX*, *YTHDC1*, *YTHDC2*, *IGF2BP1*, *IGF2BP2*, *IGF2BP3*, *YTHDF1*, *YTHDF2*, *YTHDF3*, *HNRNPA2B1*, and *HNRNPC*).^22^ Ultimately, the cor test in R programming language 3.6.1 was applied to calculate the Pearson correlation coefficient (PCC) on the expression level of m6A‐related genes and LncRNAs, thereby filtrating LncRNAs significantly associated with m6A‐related genes with standards of |Pearson *R*| > 0.3 and *p *< 0.05.

### 2.2 Screening for independent prognostic m6A‐related LncRNAs

Samples collected from TCGA were randomly classified into training set (*n* = 61) and validation set (*n* = 92) in a ratio of 4:6. Integrating with the clinical information, the univariate Cox regression analysis in R package “survival” version 2.41‐1 was implemented to filter out prognostic m6A‐related LncRNAs,^23^ and then, the multivariate Cox regression analysis was used to identify independent prognostic m6A‐related LncRNAs.

### 2.3 Prognostic score risk model based on independent prognostic m6A‐related LncRNAs

A risk model based on the prognostic coefficient and expression level of independent prognostic m6A‐related LncRNAs was established as follows:

$$\text{Prognostic score}\;\left(\text{PS}\right)=\sum {\text{Coef}}_{\text{lncRNAs}}\times {\mathrm{Exp}}_{\text{lncRNAs}}$$

Then, the TNBC samples were grouping into high risk and low risk based on the median value of PS, and the Kaplan‐Meier (KM) curve in R package was applied to evaluate the difference in survival status of patients between the two groups.^23^ The approach of support vector machine (SVM) in 6.1 e1071 Version 1.6‐8 was applied for conducting SVM classifier, thereby evaluating the prognostic performance of the PS model in TCGA validation set and GSE76250.^24^ We then employed R 3.4.1 pROC Version 1.12.1^25^ to compute the sensitivity and specificity of receiver operating characteristic (ROC) curve and to evaluate the prognostic performance of PS risk model. According to the clinical information of TNBC samples in TCGA, the univariate and multivariate Cox regression analysis in R3.6.1 survival package^23^ were used to screen independent prognostic clinical factors with log‐rank *p *< 0.05.

### 2.4 Construction of a competing endogenous RNA (ceRNA) network and pathway enrichment analysis

R3.6.1 limma version 3.34.7^26^ was used for between‐group (high‐risk vs low‐risk) difference analysis of mRNA expression matrix of samples. Differential expression genes (DEGs) were screened under the cutoff of false discovery rate (FDR) = 0.05 and |log2 fold change (FC)| = 0.263.

Human MicroRNA Disease Database (HMDD)^27^ was employed for mining TNBC‐related miRNAs while DIANA‐LncBasev2^28^ was used to establish the independent prognostic m6A‐related LncRNA‐miRNA interactions. Afterward, five databases including TargetScan Version7.2, picTar, miRanda,^29^ RNA22, and PITA^30^ were used to predict the targeted mRNAs for the miRNAs in LncRNA‐miRNA interactions, and mRNAs that appeared in at least three databases were retained. Then, mRNAs intersected with prognostic‐risk‐related DEGs were selected and were developed into miRNA‐mRNA interactions. A ceRNA network involving LncRNA‐miRNA and miRNA‐mRNA interactions was then structured and visualized by Cytoscape Version 3.6.1.^31^ Finally, we conducted Kyoto Encyclopedia of Genes and Genomes (KEGG) pathway enrichment analysis on mRNAs in the ceRNA network through DAVID version 6.8,^32^, ^33^ and *p *< 0.05 was picked as statistical significances.

## 3 RESULTS

### 3.1 Identification of m6A‐related LncRNA signature and prognostic analysis

**FIGURE 2:**
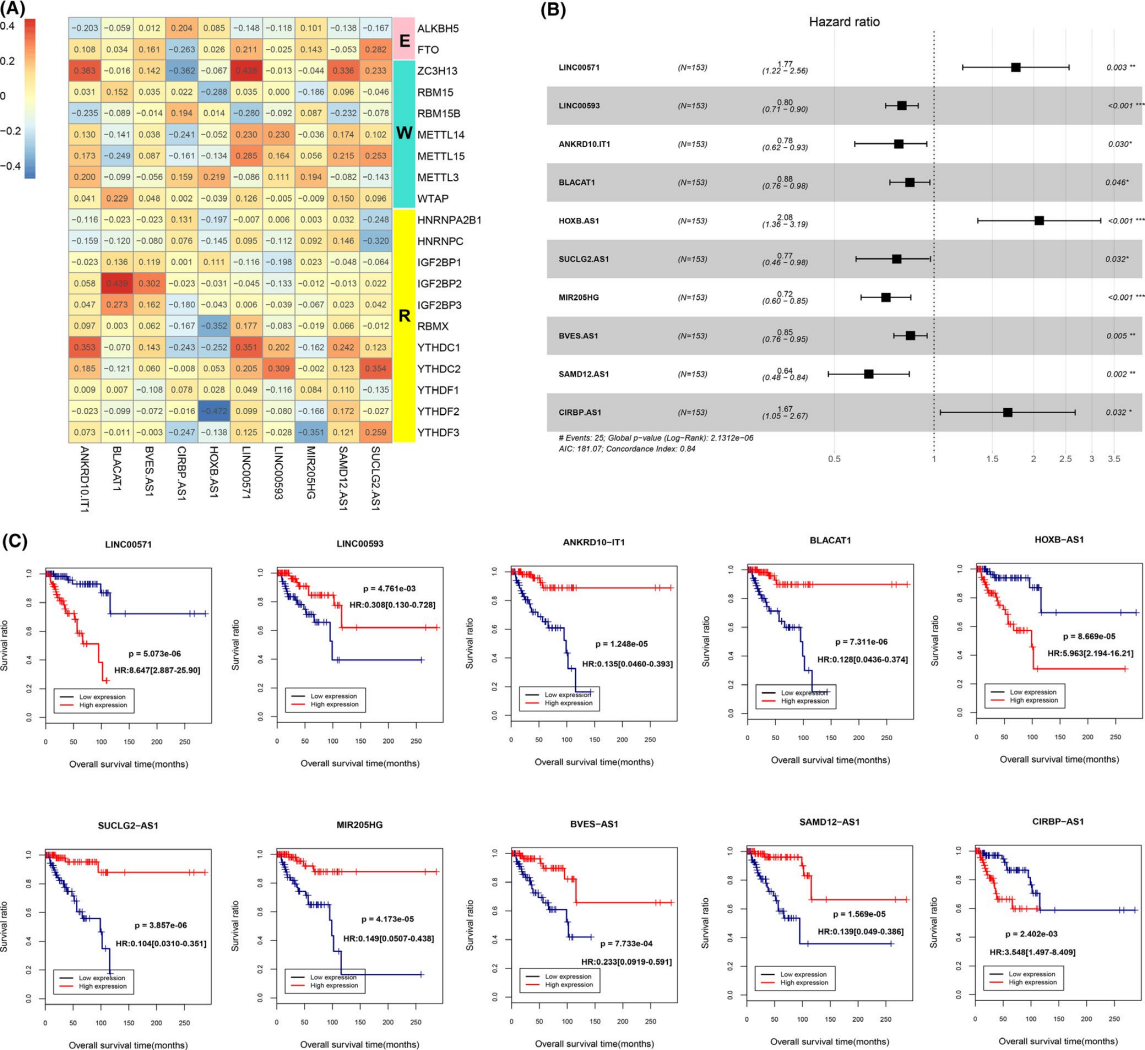
Identification of m6A‐related LncRNAs and prognostic analysis. (A) Heatmap of the associations between m6A‐related genes and the 10 independent prognostic m6A‐related LncRNAs in the expression level. (B) Forest plots showed 10 independent prognostic m6A‐related LncRNAs analyzed from the multivariate Cox regression analysis. (C) Kaplan‐Meier curves analyzed on the correlation between the expression of candidate LncRNAs and the prognosis of TNBC

By calculating the expression correlation between LncRNAs and m6A‐related mRNAs annotated in TCGA, 329 m6A‐related LncRNAs were screened out with the criteria of |Pearson *R*| > 0.3 and *p *< 0.05. Subsequently, a total of 39 LncRNAs significantly related to survival prognosis were filtrated by univariate Cox regression analysis as shown in Table S1. Ten independent prognostic m6A‐related LncRNAs including *SAMD12*‐*AS1*, *BVES*‐*AS1*, *LINC00593*, *MIR205HG*, *LINC00571*, *ANKRD10*‐*IT1*, *CIRBP*‐*AS1*, *SUCLG2*‐*AS1*, *BLACAT1*, and *HOXB*‐*AS1* were further identified by the multivariate Cox regression analysis. Their correlations with m6A‐related mRNAs in the expression level were shown in Figure 2A. The information of these ten m6A‐related prognostic LncRNAs was organized in Table 1 and was visualized in Figure 2B. The results suggested that *LINC00571*, *CIRBP*‐*AS1*, and *HOXB*‐*AS1* were found to be risk factors of TNBC patients with hazard ratio (HR) > 1, whereas *SAMD12*‐*AS1*, *BVES*‐*AS1*, *LINC00593*, *MIR205HG*, *ANKRD10*‐*IT1*, *SUCLG2*‐*AS1*, and *BLACAT1* were considered as protective factors with HR < 1. Additionally, results of the KM curve illustrated that higher expressions of *SAMD12*‐*AS1*, *BVES*‐*AS1*, *LINC00593*, *MIR205HG*, *ANKRD10*‐*IT1*, *SUCLG2*‐*AS1*, and *BLACAT1* were associated with better overall survival (OS) of TNBC (Figure 2C).

**TABLE 1:** Ten independent prognostic m6A‐related LncRNAs

Symbol	Coef	*p*–value	HR	95% CI
SAMD12‐AS1	−0.77277	1.59E‐03	0.462	0.276–0.772
BVES‐AS1	−0.29540	2.12E‐03	0.744	0.608–0.911
LINC00593	−0.30639	4.99E‐03	0.736	0.583–0.929
MIR205HG	−0.47167	6.30E‐03	0.624	0.431–0.904
LINC00571	1.06813	1.26E‐02	2.910	1.142–7.412
ANKRD10‐IT1	−1.15933	1.55E‐02	0.314	0.109–0.899
CIRBP‐AS1	1.00892	1.57E‐02	2.743	1.094–6.878
SUCLG2‐AS1	−0.97985	3.20E‐02	0.375	0.133–0.589
BLACAT1	−0.27729	4.22E‐02	0.758	0.553–0.983
HOXB‐AS1	0.76032	4.98E‐02	2.139	1.265–5.287

Note

HR > 1 indicate risk LncRNAs, and HR < 1 indicate protective LncRNAs.

Abbreviations: CI, confidence interval; HR, hazard ratio.

*p* < 0.05 indicate statistical significances.

### 3.2 Prognostic performance of risk prediction models

**FIGURE 3:**
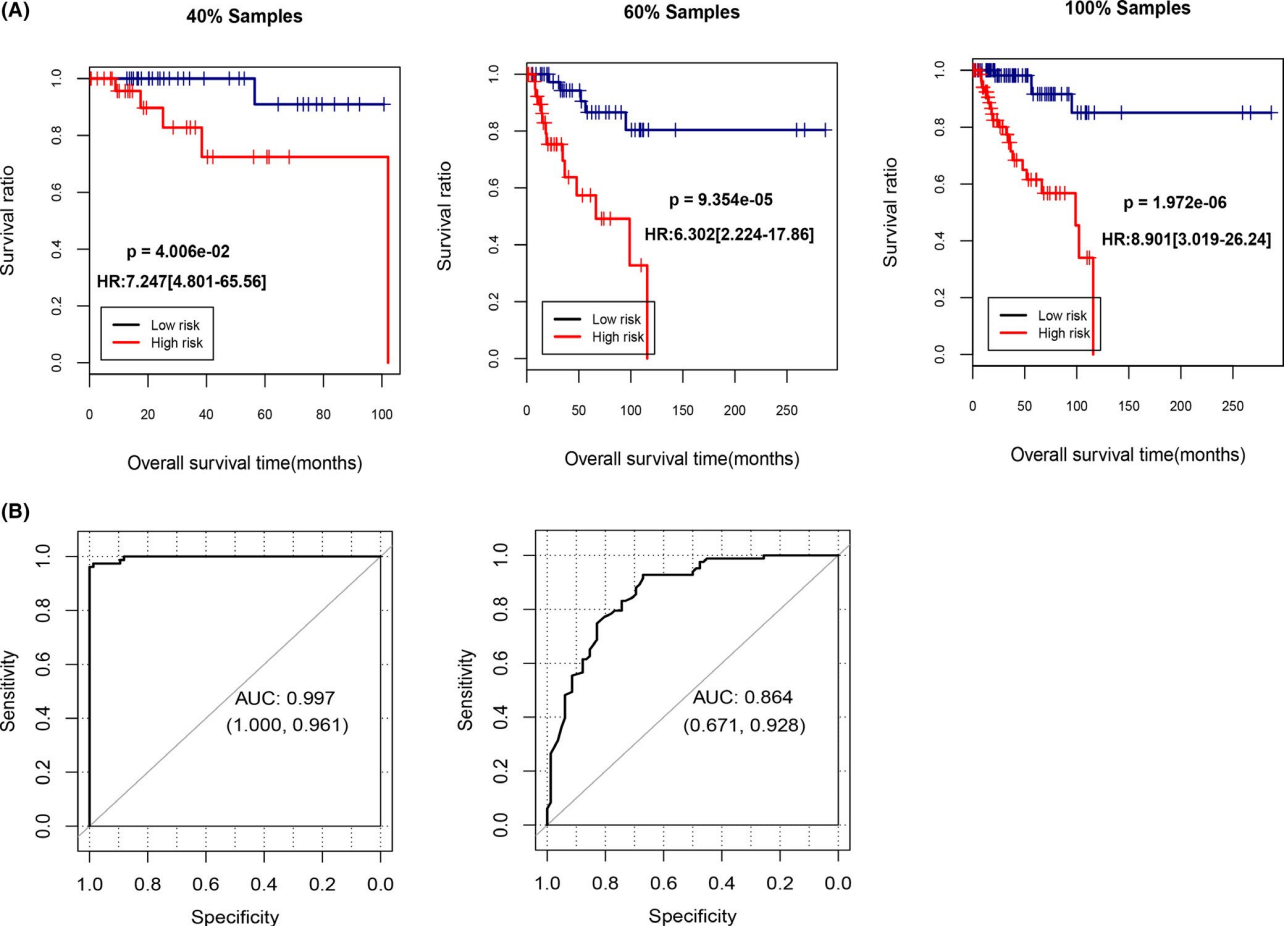
Prognostic performance of risk prediction models. (A) Kaplan‐Meier curves based on the correlation between the risk prediction model and survival prognosis in the training set, validation set, and total sample set. (B) Receiver operating characteristic (ROC) curves showed the predictive abilities of 10 LncRNAs in TCGA and GSE76250 dataset

To verify the prognostic effect of candidate LncRNAs, we established risk models. The TCGA dataset was classified into training set (*n* = 61) and validation set (*n* = 92) in a ratio of 4:6. The PS was calculated for grouping samples into high‐risk and low‐risk. KM curves (Figure 3A) analyzed from R3.6.1 survival package suggested that in all three sample sets (training set, validation set, and entire sample set), TNBC patients with lower PS had better OS status (HR > 1 and *p *< 0.05). We further analyzed the ROC curves in TCGA and GSE76250 validation sets (Figure 3B) and proved that candidate LncRNAs had excellent predictive abilities for TNBC (AUC = 0.997 and 0.864, respectively).

### 3.3 Screening for independent prognostic factors

**FIGURE 4:**
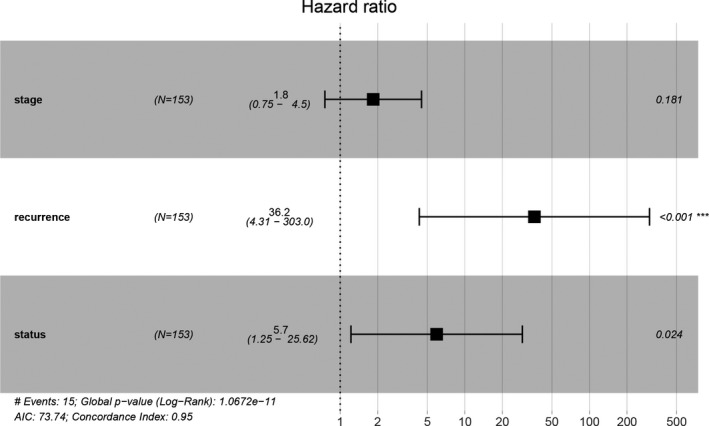
Screening for independent clinical indexes of TNBC prognosis

We continued to gather statistics on clinical characteristics of TNBC samples sourced from TCGA and performed univariate and multivariate Cox regression analysis accordingly (Figure 4; Table 2). The univariate Cox regression analysis showed that pathologic stage (HR = 4.785, 95% CI = 2.689–8.487, *p* = 5.12e‐08), recurrence (HR = 81.95, 95% CI = 10.69–627.9, *p* = 2.23e‐05) and PS model status (HR = 8.901, 95% CI = 3.019–26.24, *p* = 1.97e‐06) could affect the prognosis of TNBC patients, while the multivariate Cox regression analysis suggested recurrence (HR = 36.153, 95% CI = 4.314–303.02, *p* = 9.41e‐04) and PS model status (HR = 5.66, 95% CI = 1.251–25.62, *p* = 2.44e‐02) as independent prognostic factors of TNBC. Our results indicated that patients with lower recurrence and lower risk score had better prognoses.

**TABLE 2:** Stratification analysis of independent prognostic clinical indexes

Clinical characteristics	TCGA (*N* = 153)	Uni‐variables cox			Multi‐variables cox		
		HR	95% CI	*p*	HR	95% CI	*p*
Age (years, mean ± SD)	54.97 ± 12.11	1.003	0.972–1.035	8.64E‐01	‐	‐	**‐**
Pathologic_stage (I/II/III/IV/‐)	25/95/27/2/4	4.785	2.689–8.487	**5.12E‐08**	1.841	0.754–4.492	1.81E‐01
Radio‐therapy (Yes/No/‐)	78/60/15	0.505	0.194–1.313	1.53E‐01	‐	‐	‐
Recurrence (Yes/No/‐)	20/105/28	81.95	10.69–627.9	**2.23E‐05**	36.153	4.314–303.02	**9.41E‐04**
PS model status (High/Low)	76/77	8.901	3.019–26.24	**1.97E‐06**	5.66	1.251–25.62	**2.44E‐02**
Death (Yes/No)	25/128	‐	‐	‐	‐	‐	‐
Overall survival time (months, mean ± SD)	44.58 ± 46.21	‐	‐	**‐**	‐	‐	**‐**

Abbreviations: CI, confidence interval; HR, hazard ratio; SD, standard deviation.

Bold *p* < 0.05 indicate statistical significances.

### 3.4 Screening for DEGs between high‐risk and low‐risk groups

**FIGURE 5:**
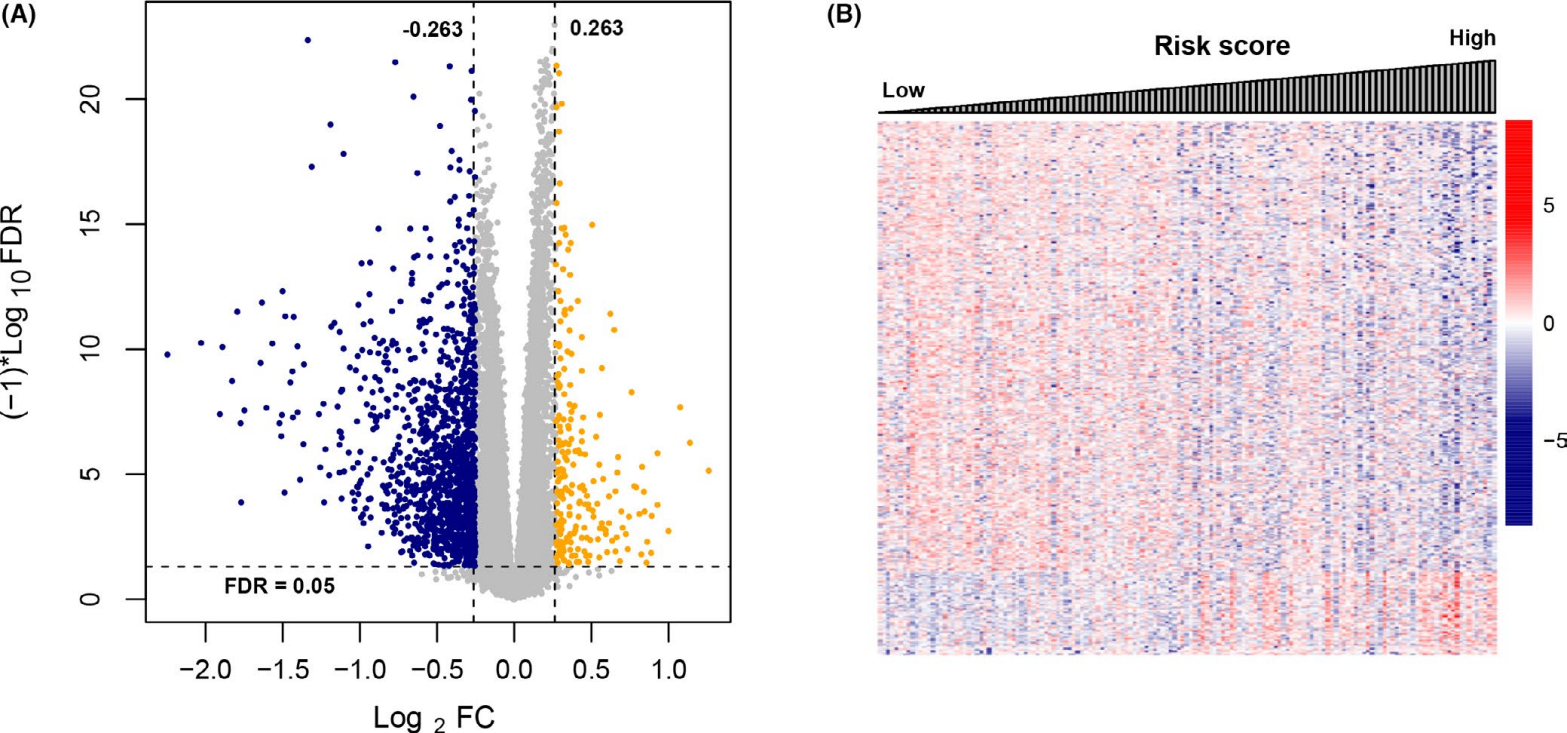
Analysis on differential expressed genes (DEGs) between high‐ and low‐risk groups. (A) Volcano plot of DEGs (The orange and the blue indicate significantly up‐regulated and down‐regulated DEGs, respectively; which are separated with the criteria of FDR > 0.05 and |log2FC| > 0.263). (B) Heatmap of DEGs expression level and related prognostic score

We used the Limma package to analysis DEGs between the high‐risk and low‐risk groups, and then obtained 814 DEGs at the cutoff of |Log_2_FC| = 0.263 and FDR = 0.05 (Figure 5A). These genes were proved to effectively distinguish samples between high‐risk and low‐risk groups (Figure 5B).

### 3.5 Construction of ceRNA network and functional analysis

**FIGURE 6:**
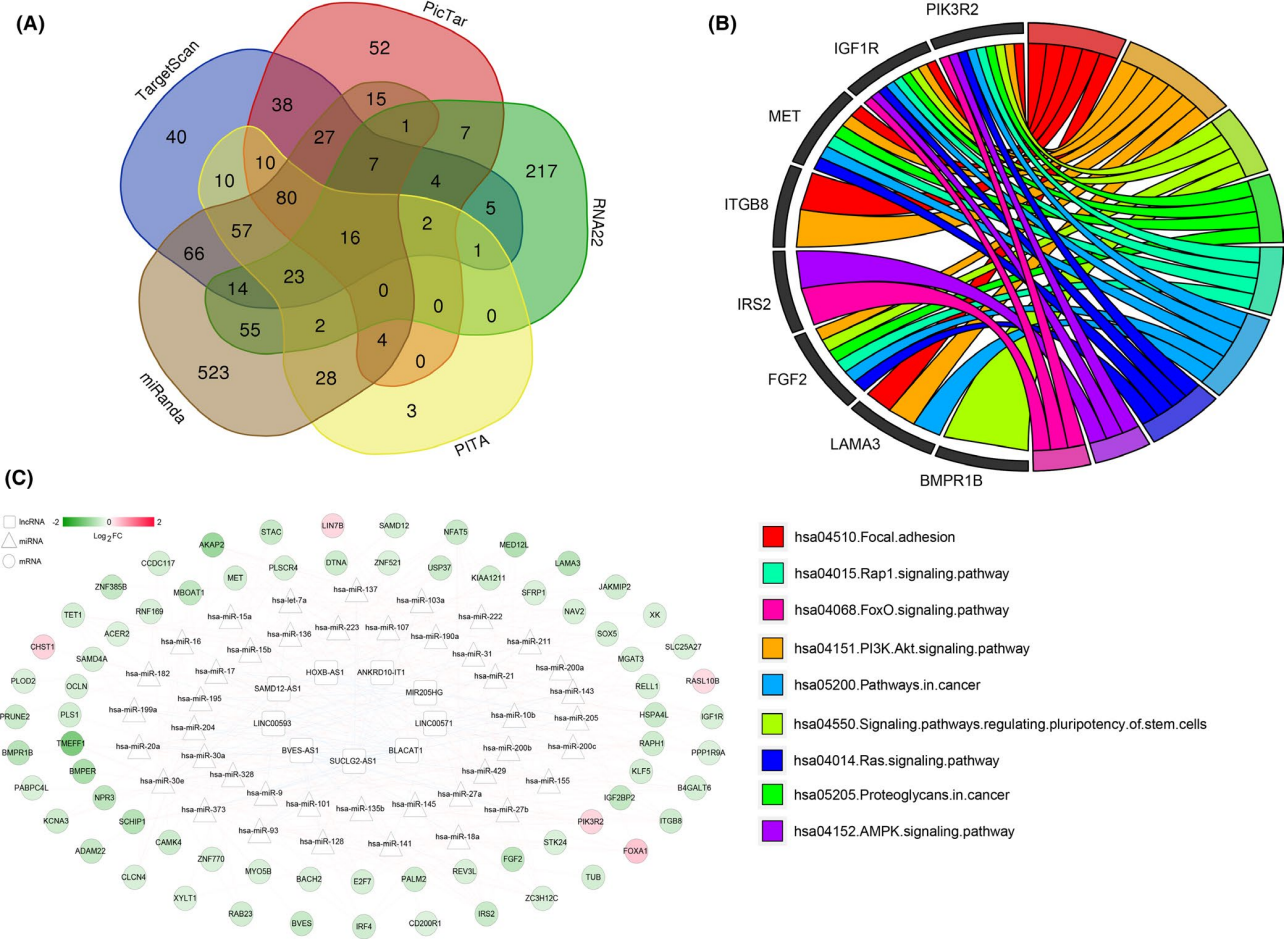
A ceRNA network establishment and functional analysis. (A) Venn diagram of target genes predicted in five databases. (B) A ceRNA regulation network (LncRNA, miRNA, and mRNA are represented by squares, triangles, and circles, respectively. The values of logFC from low to high are colored from green to red; the blue and red lines indicate LncRNA‐miRNA and miRNA‐mRNA interactions, respectively). (C) KEGG pathway enrichment of hub genes in the ceRNA network

A total of 67 miRNAs were determined to be related to TNBC in the HMDD database, then 69 interactions comprising 9 m6A‐related prognostic LncRNAs and 42 TNBC‐related miRNAs were established by DIANA‐LncBasev2. Afterward, five different databases and DEGs selected above were used to determine targeted mRNA for miRNAs in LncRNA‐miRNA interactions (Figure 6A), and finally, 249 miRNA‐mRNA interactions were obtained. By integrating LncRNA‐miRNA and miRNA‐mRNA interactions, we successfully established the ceRNA network as shown in Figure 6B. This ceRNA network incorporated 9 LncRNAs, 42 miRNAs, and 70 mRNAs, and nodes with more degrees of connections were considered to have larger contributions in this network (Table S2). Afterward, mRNAs involved in the ceRNA network were annotated and enriched by KEGG, and eight core genes and nine pathways associated with cell communication, signaling transduction, and human disease (cancer) were finally picked up (Figure 6C).

### 3.6 Expression validation

**FIGURE 7:**
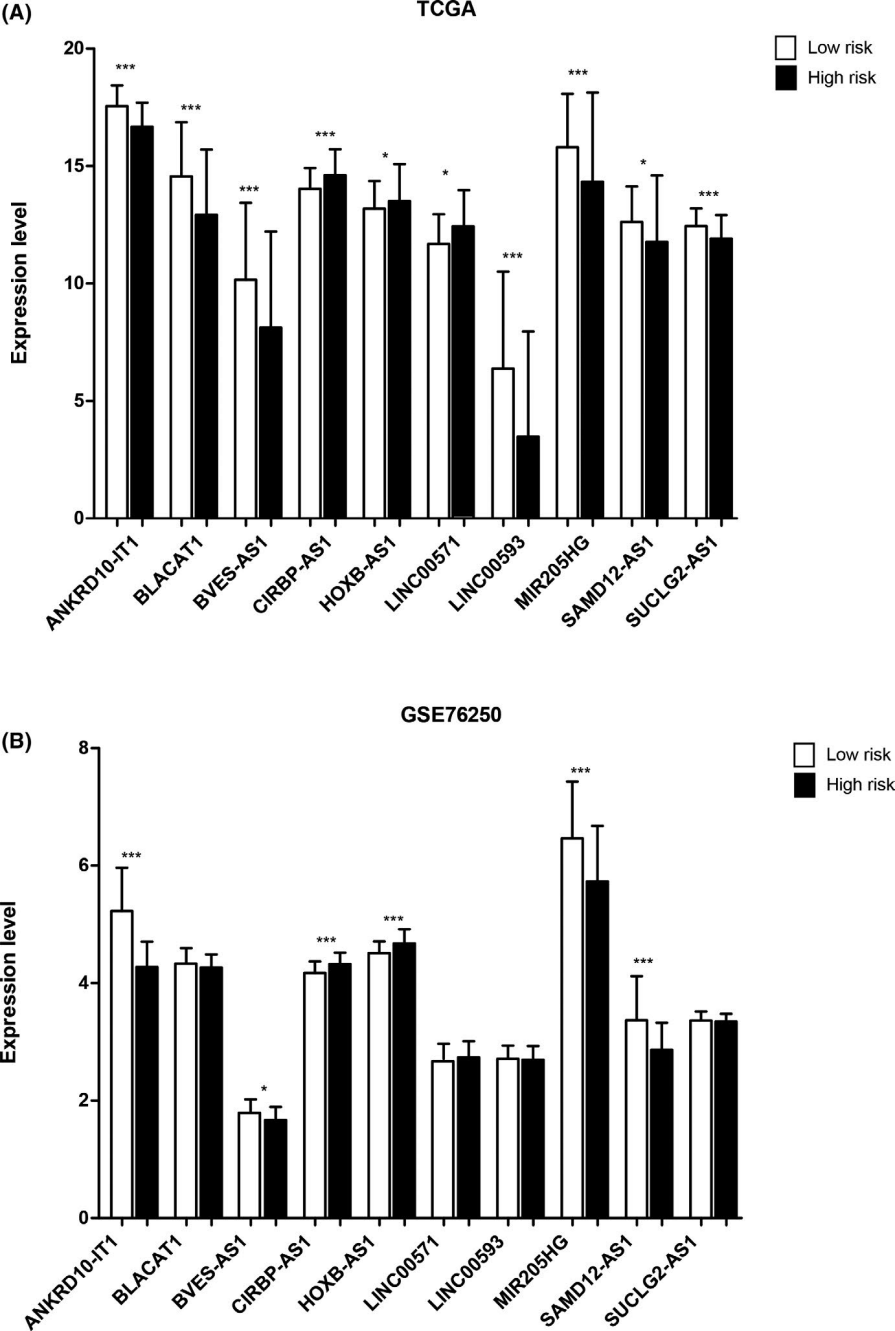
Expression validation. Expression verifications of 10 independent prognostic m6A‐related LncRNAs in TCGA (A) and GSE76250 (B), *p < 0.05, **p < 0.01, ***p < 0.001

We further extracted the expression data of 10 m6A‐related prognostic LncRNAs from TCGA and GSE76250, and then compared the expression levels between high‐risk and low‐risk groups. As shown in Figure 7A, the expressions of risk factors including *LINC00571*, *CIRBP*‐*AS1*, and *HOXB*‐*AS1* with HR > 1 were significantly higher in the high‐risk group than that in the low‐risk group, and the other protectors with HR < 1 had significantly higher expression level in the low‐risk group comparing with the high‐risk group, which were logically consistent with the prediction. Furthermore, in the GSE76250 dataset, the expression trends of candidate LncRNAs were in accordance with that in TCGA as shown in Figure 7B. Among them, the expression levels of *ANKRD10*‐*IT1*, *BVES*‐*AS1*, *CIRBP*‐*AS1*, *HOXB*‐*AS1*, *MIR205HG*, and *SAMD12*‐*AS1* were significantly different between the high‐risk and low‐risk groups.

## 4 DISCUSSION

In this study, 153 patients with TNBC were enrolled from the TCGA database according to their ER, PR, and HER‐2 status to probe into the prognostic signature of m6A‐related LncRNAs. Under the univariate and multivariate Cox regression analysis, we identified 10 m6A‐related LncRNAs (*SAMD12*‐*AS1*, *BVES*‐*AS1*, *LINC00593*, *MIR205HG*, *LINC00571*, *ANKRD10*‐*IT1*, *CIRBP*‐*AS1*, *SUCLG2*‐*AS1*, *BLACAT1*, and *HOXB*‐*AS1*) with independent prognostic values. The candidate LncRNAs‐based PS risk model could stratify TNBC patients into high‐risk and low‐risk groups, and patients with low PS risk had better survival status than high‐PS‐risk patients. ROC curves also proved that the PS risk model showed great prognostic abilities with the AUC of 0.997 and 0.864 in the TCGA validation set and GSE76250 dataset, respectively. Among these 10 LncRNAs, *ANKRD10*‐*IT1*, *BVES*‐*AS1*, *CIRBP*‐*AS1*, *HOXB*‐*AS1*, *MIR205HG*, and *SAMD12*‐*AS1* were further confirmed to have significant differences in expression level between the high‐risk and low‐risk groups. Additionally, pathway enrichment analysis on mRNAs in the ceRNA network showed that candidate LncRNAs were most likely to participate in pathways of PI3K‐AKT signaling, Rap1 signaling, and focal adhesion through LncRNA‐miRNA‐mRNA regulatory network, thus suggested a possible regulatory mechanism of m6A‐related LncRNAs on the prognosis of TNBC. To the best of our knowledge, we are the first to confirm that m6A‐related LncRNAs could be used as biomarkers to predict the survival prognosis of TNBC patients.

It was reported that *ANKRD10*‐*IT1* and *BVES*‐*AS1* were identified as prognostic signatures for hepatocellular carcinoma and colon adenocarcinoma, respectively.^34^, ^35^ Moreover, LncRNA *HOXB*‐*AS1* was determined as an oncogenic gene to be up‐regulated in endometrial cancer and to promote the proliferation, migration, and invasion of glioblastoma cells and multiple myeloma,^36^, ^37^, ^38^ while LncRNA *MIR205HG* was detected to expedite the tumor growth in esophageal squamous cell carcinoma,^39^ lung squamous cell carcinoma^40^ and cervical cancer.^41^ Additionally, LncRNA *SAMD12*‐*AS1* was proved to promote cell proliferation and to inhibit apoptosis by interacting with *NPM1* which could suppress T cell activity in TNBC by up‐regulating the transcription of *PD*‐*L1*.^42^, ^43^ Of the *BLACAT1* regulatory axis, Hu et al. proved that BLACAT1/miR‐150‐5p/CCR2 could promote the cell proliferation and metastasis of breast cancer.^44^ The above studies summarized the correlations between candidate LncRNAs and TNBC‐related diseases, and partially confirmed the reliability of our current results.

Considering the important roles of these LncRNAs, we further investigated their underlying regulatory mechanism. Through the network construction of ceRNA crosstalk and pathway enrichment of related mRNAs, *PIK3R2* was identified as core genes to involve in pathways of cell communication, signaling transduction, and cancers through regulations of BVES‐AS1/miR135b, BLACAT1/miR‐30, SAMD12‐AS1/miR‐30, and MIR205HG/miR‐30 axes. Khoury et al. have proved that breast cancer patients had 0.08% of *PIK3R2* mutation in the PI3K‐Akt signaling pathway.^45^ PI3K regulatory subunit *PIK3R2* could also be modulated by *IRF6* through the PI3K‐Akt pathway to control the pathogenesis of breast cancer.^46^ Based on previous studies, *SAMD12*‐*AS1* was considered to promote cell proliferation through the interaction with *NPM1*, while abnormal regulation of *BLACAT1* could also promote the proliferation and metastasis of cancer cells.^43^, ^47^ Moreover, miR‐30 was proved by Bao et al. to mediate cell invasion and metastasis in breast cancer.^48^ Combined with our predictions, we hypothesized that BLACAT1/miR‐30/PIK3R2 and SAMD12‐AS1/miR‐30/PIK3R2 axes could mediate the regulation of TNBC through the PI3K‐Akt signaling pathway, but the follow‐up experiments are still needed for verification.

However, it is considerable that the limitation of sample size, the lack of clinical information of samples, and the absence of detection on methylation level of candidate LncRNAs are shortcomings in this study. Therefore, a larger sample size and the collection of solid tumor samples are required in the following studies to investigate the regulatory mechanism of candidate LncRNAs in TNBC. Moreover, we will further explore the differences in the prognostic effect of m6A‐related LncRNAs on TNBC in various populations for a more accurate prognosis treatment strategy.

Conclusively, by mining the data from public databases, we identified ten m6A‐related prognostic LncRNA signatures, confirmed their predictive roles in prognostic risk for TNBC patients, and pointed out the potential mechanisms of candidate LncRNA‐related ceRNA regulation. Our findings may help to improve the prognosis for patients with TNBC.

## CONFLICT OF INTEREST

The authors declare that they have no conflict of interest.

## AUTHOR CONTRIBUTION

JW and ML involved in conception and design of the research, acquisition of data, and drafting the manuscript. JW, YC, and GZ involved in analysis and interpretation of data. YC and GZ involved in statistical analysis. YC, GZ, and ML involved in funding. JW involved in revision of manuscript for important intellectual content. All authors read and approved the final manuscript.

## Supporting information

Table S1‐S2

## Data Availability

In this study, the data from TCGA database are available from https://gdc‐portal.nci.nih.gov/, while the GSE76250 dataset can be found in NCBI‐GEO (http://www.ncbi.nlm.nih.gov/geo/).
