# Supplementary material for: A ten N6‐methyladenosine‐related long non‐coding RNAs signature predicts prognosis of triple‐negative breast cancer
Source: J Clin Lab Anal. 2021 May 2;35(6):e23779. doi: 10.1002/jcla.23779 (PMC8183938; doi:10.1002/jcla.23779)
Supplement: Supplementary file 1 — Table S1‐S2 [file JCLA-35-e23779-s001.docx]

**Supplemental Table 1** 39 LncRNAs significantly related to survival prognosis of TNBC analyzed by univariate Cox regression analysis

| **Symbol** | **coef** | **exp(coef)** | **se(coef)** | **z** | ***P*** |
| --- | --- | --- | --- | --- | --- |
| FMR1-IT1 | -0.225 | 0.798 | 0.0554 | -4.07 | 2.35E-05 |
| AP4B1-AS1 | -0.227 | 0.797 | 0.068 | -3.34 | 4.20E-04 |
| TRAPPC12-AS1 | -0.219 | 0.803 | 0.0742 | -2.96 | 1.55E-03 |
| LINC01359 | -0.182 | 0.834 | 0.07 | -2.6 | 4.65E-03 |
| KIRREL3-AS1 | -0.0743 | 0.928 | 0.0287 | -2.58 | 4.90E-03 |
| LINC00571 | -0.216 | 0.806 | 0.0839 | -2.57 | 5.00E-03 |
| LINC00593 | -0.117 | 0.89 | 0.0462 | -2.52 | 6.00E-03 |
| NARF-IT1 | -0.167 | 0.846 | 0.0666 | -2.51 | 6.00E-03 |
| IDI2-AS1 | -0.163 | 0.85 | 0.0655 | -2.49 | 6.50E-03 |
| OCIAD1-AS1 | -0.198 | 0.82 | 0.085 | -2.33 | 1.00E-02 |
| TEX41 | 0.352 | 1.42 | 0.153 | 2.31 | 1.05E-02 |
| LINC01122 | -0.129 | 0.879 | 0.0575 | -2.25 | 1.20E-02 |
| LINC01424 | -0.139 | 0.871 | 0.0623 | -2.22 | 1.30E-02 |
| DNM3OS | 0.242 | 1.27 | 0.112 | 2.16 | 1.55E-02 |
| LINC01116 | 0.273 | 1.31 | 0.126 | 2.16 | 1.55E-02 |
| LINC01504 | 0.36 | 1.43 | 0.175 | 2.05 | 2.00E-02 |
| CBR3-AS1 | 0.358 | 1.43 | 0.178 | 2.02 | 2.20E-02 |
| BANCR | -0.0771 | 0.926 | 0.0386 | -2 | 2.30E-02 |
| ANKRD10-IT1 | -0.404 | 0.668 | 0.204 | -1.98 | 2.35E-02 |
| BLACAT1 | -0.0953 | 0.909 | 0.0483 | -1.97 | 2.45E-02 |
| HCP5 | -0.319 | 0.727 | 0.162 | -1.97 | 2.45E-02 |
| HOXB-AS1 | 0.31 | 1.36 | 0.158 | 1.96 | 2.45E-02 |
| SUCLG2-AS1 | -0.409 | 0.665 | 0.208 | -1.96 | 2.50E-02 |
| MIR205HG | -0.131 | 0.877 | 0.0677 | -1.94 | 2.65E-02 |
| BVES-AS1 | -0.0905 | 0.913 | 0.047 | -1.92 | 2.70E-02 |
| LINC01347 | -0.196 | 0.822 | 0.103 | -1.91 | 2.80E-02 |
| YTHDF3-AS1 | 0.339 | 1.4 | 0.179 | 1.9 | 2.90E-02 |
| ADAMTSL4-AS1 | 0.376 | 1.46 | 0.203 | 1.86 | 3.15E-02 |
| SNRK-AS1 | -0.103 | 0.902 | 0.0555 | -1.85 | 3.20E-02 |
| SAMD12-AS1 | -0.157 | 0.855 | 0.0852 | -1.84 | 3.30E-02 |
| CIRBP-AS1 | 0.331 | 1.39 | 0.187 | 1.77 | 3.85E-02 |
| NNT-AS1 | -0.373 | 0.689 | 0.215 | -1.74 | 4.10E-02 |
| PAXIP1-AS2 | -0.445 | 0.641 | 0.26 | -1.71 | 4.30E-02 |
| ZNF32-AS2 | -0.163 | 0.85 | 0.0952 | -1.71 | 4.35E-02 |
| LINC00954 | -0.179 | 0.836 | 0.105 | -1.71 | 4.40E-02 |
| HCG27 | -0.325 | 0.722 | 0.191 | -1.7 | 4.45E-02 |
| DIRC3-AS1 | -0.0649 | 0.937 | 0.0383 | -1.69 | 4.50E-02 |
| MIR22HG | 0.41 | 1.51 | 0.244 | 1.68 | 4.65E-02 |
| NBAT1 | -0.093 | 0.911 | 0.0558 | -1.66 | 4.80E-02 |

**Supplemental Table 2** Hub LncRNAs, miRNAs and mRNAs with more node degrees

| **Type** | **Term** | **Degree** | **Betweenness** | **Closeness** |
| --- | --- | --- | --- | --- |
| LncRNA | BLACAT1 | 18 | 2920.676 | 0.42553192 |
| LncRNA | SUCLG2-AS1 | 13 | 1054.769 | 0.3529412 |
| LncRNA | BVES-AS1 | 9 | 1064.9916 | 0.3409091 |
| LncRNA | MIR205HG | 8 | 736.18896 | 0.36585367 |
| LncRNA | HOXB-AS1 | 5 | 230.89809 | 0.2739726 |
| LncRNA | LINC00571 | 5 | 183.18903 | 0.2857143 |
| LncRNA | LINC00593 | 5 | 176.29083 | 0.32085562 |
| LncRNA | SAMD12-AS1 | 5 | 346.9862 | 0.32258064 |
| miRNA | hsa-miR-30a | 25 | 2724.5828 | 0.41237113 |
| miRNA | hsa-miR-30e | 18 | 1019.4092 | 0.36923078 |
| miRNA | hsa-miR-17 | 16 | 791.37427 | 0.3883495 |
| miRNA | hsa-miR-20a | 16 | 907.6917 | 0.38338658 |
| miRNA | hsa-miR-93 | 16 | 858.12646 | 0.39087948 |
| miRNA | hsa-miR-373 | 12 | 605.6481 | 0.36474165 |
| miRNA | hsa-let-7a | 11 | 1369.0646 | 0.3478261 |
| miRNA | hsa-miR-27a | 10 | 443.26804 | 0.3478261 |
| miRNA | hsa-miR-27b | 10 | 458.2455 | 0.34985423 |
| mRNA | IRS2 | 12 | 1294.4795 | 0.3846154 |
| mRNA | E2F7 | 11 | 757.7795 | 0.3726708 |
| mRNA | NFAT5 | 11 | 794.2663 | 0.37037036 |
| mRNA | AKAP2 | 10 | 882.902 | 0.3243243 |
| mRNA | ZC3H12C | 8 | 291.71472 | 0.3314917 |
| mRNA | NAV2 | 8 | 307.35898 | 0.33519554 |

LncRNA, long non-coding RNA; miRNA, microRNA.
